# New Scanographic Index for the Detection of Frailty in Patients with Cirrhosis with a Prognostic Impact

**DOI:** 10.34172/mejdd.2024.376

**Published:** 2024-04-30

**Authors:** Christele El Khoueiry, Rita Slim, Mohammad Rida, Bernard Khoury, Khalil Honein, Tarek Smayra, Cesar Yaghi

**Affiliations:** ^1^Hotel Dieu de France Hospital, Beirut, Lebanon

**Keywords:** Cirrhosis, Frailty, Score, Prognostic

## Abstract

**Background:**

Frailty is linked to an increased incidence of hepatic decompensation and mortality in cirrhosis. The aim of our study was to identify a novel scanographic score that predicts frailty and its impact in cirrhosis.

**Methods:**

This study included 51 patients with cirrhosis. We used the frailty scale risk assessment score to identify frail patients. The density and area of different muscles at L3 level were analyzed on computed tomography (CT) sections. The L3 skeletal muscle area adjusted to height and density ratio (L3-SMDHR) was defined as L3 muscle wall*height/density.

**Results:**

The L3-SMHDR is significantly higher in frail patients and in patients with Child B/C scores. Frailty was correlated with L3-SMHDR. Frailty and L3- SMHDR were correlated with liver-related events (LRE). We set the most appropriate cut-offs of L3-SMHDR for both sensitivity and specificity by using the ROC: 5.4 for males and 4.7 for females. The AUROC score was 0.784 for male and 0.975 for female patients. The Kappa score between frailty and L3-SMHDR was 0.752, with a percentage of agreement of 87.5%, showing a substantial agreement. This ratio with the divided categories has a sensitivity of 100%, a specificity of 76%, a positive predictive value of 79.3% and a negative predictive value of 100%. Patients with high L3-SMHDR have significantly lower survival time and a higher incidence of LRE.

**Conclusion:**

The L3-SMHDR is a new index for identifying frailty in cirrhosis by using measurable and reproducible variables. It can be used as a prognostic factor for frailty in patients with cirrhosis.

## Introduction

 The concept of frailty in patients with cirrhosis is defined as a state of increased vulnerability resulting from a decline in functional abilities and reduced physiological reserve. It is believed to be present in half of patients with advanced liver disease, but its prevalence may be variable according to etiology^1^ and because of complex interacting factors including sex, age, disease etiology, disease severity, complications, and nutritional status.^2^ The diagnosis of frailty in patients with cirrhosis is essential because it is linked to a higher incidence of hepatic decompensation, hospitalization, transplant delisting, post-transplant complications, and an increased mortality.^3^ Moreover, it can be a reversible state when detected early. Cirrhotic patients with frailty present a higher incidence of ascites (57% vs 34%), lower levels of serum albumin (31.2 g/L vs 35.1 g/L), higher risk of hepatic encephalopathy (26% vs 17%), and consequently increased number of hospitalizations.^4,5^ Furthermore, frailty in patients with cirrhosis is significantly associated with depression.^6^ Despite its prognostic implication, frailty is seldom sought in patients with cirrhosis.

 Several scoring systems are used to diagnose frailty. Different tools include gait speed, hand grip strength (HGS), chair stands, balance, reported fatigue, and functional ability. Fried Frailty Index (FFI) defines frailty as the presence of three or more criteria: weight loss, self-reported exhaustion, loss of skeletal muscle function, slow walking speed and low physical activity.^7^ FFI is validated for the prediction of mortality in patients with cirrhosis with one point increase in the score leads to an increase of 50% mortality. FFI may predict the mortality risk in patients with MELD scores < 18 in whom mortality is underestimated. The clinical frailty scale (CFS) is based only on clinical judgment and is easy to use in daily practice. It is a score from 1 (very fit) to 9 (terminally fit). CFS is a predictor of increased mortality in outpatient patients with cirrhosis regardless of muscle mass (hazard ratio 1.534, *P* = 0.007).^8^ For the hospital inpatients, a CFS higher than 4, was an independent predictor for the 28 days mortality (mortality of 30.7% for hospital inpatients with a CFS higher than 4 and 19.1% for those with a CFS lower than 4).^8^ A high CFS is associated with increased rates of unplanned hospitalization (57% frail vs. 24% not frail, adjusted odds ratio 3.6, *P* = 0.0008), high risk of acute kidney injury (3.7-fold risk increase with a CFS higher than 4), or death.^7,9,10^ The liver frailty index (LFI) includes the sex-adjusted HGS, chair stands, and balance. Frailty is defined by a score ≥ 4.5, and prefrailty between 3.2 and 4.4.^11^ It has an equivalent ability to estimate the three-month waitlist mortality as MELD-Na score.^12^ The Rockwood Frailty Index contains 70 items related to comorbidities, neurological signs and changes in physical functioning. It is associated with mortality, prolongation of the hospital stay and readmission in inpatients with cirrhosis.^2^ The Braden Scale is a marker of frailty that has been shown to predict the 90-day mortality, the length of stay and the discharge to rehabilitation in hospitalized patients with cirrhosis.^13^ The frailty scale risk assessment score measures fatigue, resistance, ambulation, illness, and loss of weight. Patients having a score ≥ 3 are classified as frail.^14^ It is validated in patients undergoing chronic dialysis and is associated with the probability of having dialysis-related complications.^15^

 The sarcopenia is defined as the loss of muscle mass, strength, and function. It is prevalent in patients with cirrhosis (40%-70%) and varies according to etiologies. It is associated with an increased risk of morbidity and mortality.^16,17^ Malnutrition and malabsorption of fats and fat-soluble vitamins, with reduced total energy intake, are linked to sarcopenia in cirrhosis. Patients with cirrhosis and sarcopenia have a lower quality of life due to the loss of muscle mass or due to the increased risks of sepsis and other complications of cirrhosis.^18^ There is no established gold standard for the diagnosis of sarcopenia in patients with cirrhosis. The HGS is a good indicator of the functional muscle strength of the hand and forearm. MRI is an objective measure of the loss of lower limb muscle. However, it is expensive with a limited access technique. Computed tomography (CT) scan is a highly accurate and readily available technique. The CT L3 skeletal muscle index is now the most used to evaluate sarcopenia in patients with cirrhosis. The diagnostic cut-offs are extrapolated from oncology patients: < 38.5 cm/m^2^ for women and < 52.4 cm/m^2^ for men.^16^ The ascites and edema in patients with cirrhosis do not seem to affect the skeletal muscle index, especially if combined with the HGS.^19^

 In both cirrhosis and frailty, we note a state of chronic inflammation (after the elevation of TNF-α and interleukin-6) that can lead to the disturbance of the homeostatic balance between myocyte production, destruction and hypertrophy and thereafter, muscle breakdown.^2^ Therefore, the destruction of the muscle is accompanied by higher fat content in the muscle, higher muscle wall area, and lower density on the CT scan; thus, we talk about myosteatosis. It is present in nearly half of the patients with cirrhosis, and it does not necessarily occur with the loss of muscle mass at the same time.^20^ CT is now a common modality for the diagnosis of myosteatosis in patients with cirrhosis.^21^

 The aim of our study was to identify a novel scanographic score to predict frailty and its impact, in patients with cirrhosis.

## Methods

###  Study Population

 This is a cross-sectional study that included 51 patients with cirrhosis enrolled between April 2020 and June 2022. These patients were either inpatient or outpatient. The study was conducted at the Hotel Dieu de France Hospital in Beirut. Patients were eligible if they were 18 years old or older and were diagnosed with cirrhosis of any etiology. Patients with neurological or psychiatric diseases and severe cardiac or renal insufficiency were excluded from the study.

###  Data Collection

 The patients filled out a questionnaire containing the demographic and anthropometric characteristics (body mass index [BMI], age, sex), the etiology and complications of the cirrhosis (ascites, encephalopathy, Child-Pugh score and the MELD sodium), and the latest blood test (creatinine, albumin, bilirubin, sodium, international normalized ratio). The patients were classified into two groups: frail and not frail, using the frailty scale risk assessment score.

 Several sections of CT scan of the patients were analyzed on Coreslicer® to measure the average total muscle density (HU) and the area of the different muscles and fat at L3 level. The IMAC (intramuscular adipose tissue content) is defined as the paravertebral muscle/subcutaneous fat tissue attenuation ratio. The L3 skeletal muscle index (L3 SMI) was calculated by dividing the muscle wall at L3 level by the height squared. The L3 density-related muscle index (L3-DRMI) was calculated by dividing the L3 SMI by the density. The L3 skeletal muscle area adjusted to height and density ratio (L3-SMHDR) was defined as L3 muscle wall*height /density.

###  Statistical Analysis

 All data collected from the questionnaires were introduced in a table and analyzed using Statistical Package for Social Sciences (SPSS) software. Continuous variables were summarized as mean and standard deviation. The categorical variables were reported as numbers and percentages. The *t* test was used to assess the differences between groups for the continuous variables and the Pearson chi-square test for the categorical variables.The *t* test was used to assess the association of the different parameters of the CT scans with respect to the frailty status and the Child-Pugh score, depending on the sex of the cirrhotic patient. A *P* ≤ 0.05 was used to denote statistical significance. The Pearson correlation coefficient was used to seek a relationship between different components of CT findings, liver-related events (LRE), and liver function. We used the area under the receiver operating characteristic curve to determine the most appropriate cut-off of L3-SMHDR for best sensitivity and specificity. Survival analysis was done using the Kaplan-Meier method and Log-rank to compare between groups.

 The study was conducted in accordance with the Declaration of Helsinki, and the protocol of the study was approved by the Ethics Committee of Saint Joseph University of Beirut and Hotel Dieu de France Hospital (CEHDF 1755). Each patient signed written informed consent.

## Results

###  Population Characteristics 

 Our study included 51 patients with cirrhosis (aged 65 ± 12.7 years), including 32 men (62.7%) and 19 women (37.3%). The MELD Na score in the cirrhotic group ranged from 7 to 39, with a mean of 18.1 ± 7.78 SD. Of the patients with cirrhosis: 45.1% were classified as frail, 64.7% had portal hypertension (HTP) related events, and 72.5% had LRE. 37.3% of the patients with cirrhosis died during the period of the data collection.

###  Description of CT Parameters

**Figure 1:**
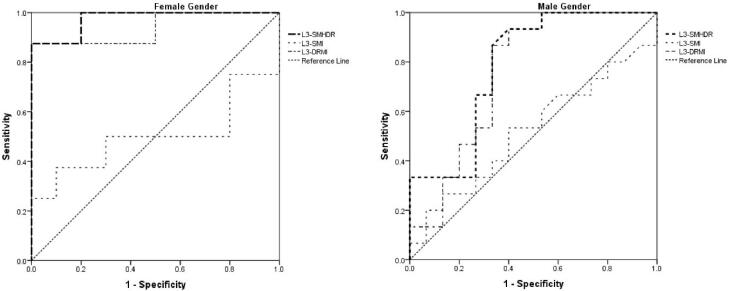
The L3-SMHDR in frail and non-frail patients depending on sex

 Various measurements of the CT sections, including the measurement of the area and the density of different muscles at different levels, are shown in Table 1. The mean total muscle density is significantly lower in frail patients than in non-frail patients in both male and female groups. The intramuscular adipose tissue content is significantly higher in the frail group compared with the non-frail group in both male and female groups. The L3-DRMI is significantly higher in the frail group compared with the non-frail group in both male and female groups. Moreover, the L3-SMHDR was significantly higher in the frail group compared with the non-frail group in both male and female groups (Figure 1).

**Table 1:** The various measurements of CT sections of patients with cirrhosis stratified by frailty status

	**Men**			**Women**		
	**Not Frail (n=15)**	**Frail (n=15)**	***P***	**Not Frail (n=11)**	**Frail (n=8)**	***P***
L3 muscle wall (cm^2^)	141.4 ± 33.8	162.7 ± 47.8	0.153	112.8 ± 25.2	127.6 ± 36.4	0.308
L3 SC FAT (cm^2^)	153.9 ± 63.3	148.9 ± 70.4	0.834	252.8 ± 149.9	212.3 ± 122.8	0.539
L3 VIS FAT (cm^2^)	184.7 ± 79.5	190.3 ± 96.6	0.861	136.9 ± 90.2	132.3 ± 33.6	0.892
Average muscle density (HU)	48 ± 8.3	40.1 ± 10	0.021	50.5 ± 13.3	35.5 ± 10.1	0.016
IMAC	-0.71 ± 0.47	-0.27 ± 0.46	0.012	-0.64 ± 0.50	-0.13 ± 0.35	0.025
L3 SMI (cm^2^/m^2^)	50.1 ± 13.6	55.3 ± 12.3	0.34	43.4 ± 9.9	47.3 ± 13.8	0.492
L3-SMHDR (cm^2^.m/HU)	5.4 ± 1.8	7.8 ± 2.3	0.004	3.7 ± 1.1	6.6 ± 0.9	< 0.001
L3-DRMI (cm^2^/m^2^.HU)	1.10 ± 0.41	1.43 ± 0.46	0.047	0.96 ± 0.48	1.35 ± 0.26	0.05

Mean ± Standard deviation, *P* value significance ≤ 0.05 SC: subcutaneous, VIS: visceral, IMAC: intramuscular adipose tissue content, SMI: skeletal muscle index, DRMI: density-related muscle index, SMHDR: skeletal muscle area adjusted to height and density ratio.

 The mean total muscle density is significantly lower in patients with Child B/C cirrhosis compared with the Child A patients in both the male and female groups (Table 2). The ratio of the L3 skeletal muscle index to the density and the ratio of L3 muscle wall*height /density were significantly higher in patients with Child B/C cirrhosis compared with Child A patients in both the male and female groups.

**Table 2:** Various measurements of CT sections of patients with cirrhosis stratified by the Child-Pugh score

	**Men**			**Women**		
	**Child-Pugh A** **(n=8)**	**Child-Pugh B/C** **(n=22)**	***P*** ** value**	**Child-Pugh A** **(n=9)**	**Child-Pugh B/C** **(n=10)**	***P *** **value**
L3 muscle wall (cm^2^)	132.9 ± 35.9	158.6 ± 42.3	0.118	121.1 ± 35.2	117.2 ± 27.1	0.788
L3 SC FAT (cm^2^)	174.6 ± 65	142.6 ± 65.1	0.221	293.4 ± 175.3	183.8 ± 62.3	0.081
L3 VIS FAT (cm^2^)	236.7 ± 49.4	168 ± 92.3	0.044	146.1 ± 97.4	124.9 ± 35.5	0.528
Average total muscle density (HU)	49.9 ± 7.7	42.1 ± 9.9	0.043	54 ± 8	35.3 ± 12.3	0.001
IMAC	-0.67 ± 0.5	-0.43 ± 0.51	0.252	-0.67 ± 0.5	-0.2 ± 0.42	0.041
L3 SMI (cm^2^/m^2^)	46.1 ± 13.3	55.1 ± 14.4	0.134	45.6 ± 13.3	44.6 ± 10.4	0.854
L3-DRMI (cm^2^/m^2^.HU)	0.95 ± 0.27	1.38 ± 0.47	0.021	0.87 ± 0.29	1.36 ± 0.43	0.010
L3-SMHDR (cm^2^.m/HU)	4.69 ± 1.25	7.26 ± 2.30	0.006	4 ± 1.46	5.98 ± 1.56	0.013

Mean ± Standard deviation, *P* value significance ≤ 0.05 SC: subcutaneous, VIS: visceral, IMAC: intramuscular adipose tissue content, SMI: skeletal muscle index, DRMI: density-related muscle index, SMHDR: skeletal muscle area adjusted to height and density ratio.

###  Correlation of Frailty and SMHDR with Liver-Related Events

 We performed a Pearson correlation between CT-measured parameters and the presence of frailty as well as the occurrence of LRE and HTP-related events. Skeletal muscle index was significantly correlated with muscle wall area as well as both visceral and subcutaneous fat area, but not with frailty. Frailty was inversely correlated with average muscle density and correlated with L3-SMHDR and L3-DRMI. In the same manner, frailty and SMHDR were correlated with LRE (Table 3).

**Table 3:** Correlations between liver-related events, portal hypertension-related events, frailty, and different CT-measured parameters

	**L3 Muscle Wall (cm^2^)**	**L3 SC FAT (cm^2^)**	**L3 VIS FAT (cm^2^)**	**Average total muscle density** **(HU)**	**IMAC**	**SMI** **(cm^2^/m^2^)**	**L3-SMHDR** **(cm^2^.m/HU)**	**L3-DRMI** **(cm^2^/m^2^.HU)**	**Frail**	**HTP related events**
L3 SC FAT (cm^2^)	0.242 (0.027)									
L3 VIS FAT (cm^2^)	0.337 (0.002)	0.402 ( < 0.001)								
Mean muscle density (HU)	0.016 (0.883)	0.155 (0.158)	-0.236 (0.031)							
IMAC	-0.091 (0.418)	0.137 (0.221)	0.279 (0.011)	-0.677 ( < 0.001)						
SMI (cm^2^/m^2^)	0.801 ( < 0.001)	0.477 (0.001)	0.301 (0.038)	0.147 (0.317)	-0.107 (0.469)					
L3-SMHDR (cm^2^.m/HU)	0.556 ( < 0.001)	-0.056 (0.707)	0.134 (0.362)	-0.638 ( < 0.001)	0.436 (0.002)	0.261 (0.073)				
L3-DRMI (cm^2^/m^2^.HU)	0.508 ( < 0.001)	0.113 (0.439)	0.151 (0.3)	-0.667 ( < 0.001)	0.511 ( < 0.001)	0.445 (0.002)	0.882 ( < 0.001)			
Frail	0.183 (0.198)	-0.098 (0.495)	-0.062 (0.668)	-0.462 (0.001)	0.46 (0.001)	0.032 (0.831)	0.602 ( < 0.001)	0.526 ( < 0.001)		
HTP related events	0.126 (0.384)	0.012 (0.936)	-0.064 (0.657)	-0.19 (0.185)	0.155 (0.281)	0.136 (0.363)	0.167 (0.261)	0.214 (0.145)	0.211 (0.141)	
LR Event	0.081 (0.578)	-0.318 (0.025)	-0.349 (0.013)	-0.414 (0.003)	0.276 (0.052)	0.05 (0.737)	0.437 (0.002)	0.41 (0.004)	0.456 (0.001)	0.469 (0.001)

Correlation coefficient (P value), *P* value significance ≤ 0.05. SC: subcutaneous, VIS: visceral, IMAC: intramuscular adipose tissue content, SMI: skeletal muscle index, DRMI: density-related muscle index, SMHDR: skeletal muscle area adjusted to height and density ratio, HTP: portal hypertension, LR: liver-related.

###  L3-SMHDR Cut-offs for Predicting Frailty

 We used the ROC to determine the most appropriate cut-off of L3-SMHDR for both sensitivity (Se) and specificity (Sp). The AUROC score was 0.784 in the male group and 0.975 in the female group. The determined cut-offs were 5.4 for men (Se = 80% Sp = 67%) and 4.7 for women (Se = 87%, Sp = 80%) (Table 4).

**Table 4:** The area under the curve of L3 SMI, L3-DRMI and L3-SMHDR depending on the sex

**Sex**	**Test result variable(s)**	**Area under the curve (95% CI)**
Male	L3-SMHDR	0.784 (0.615 - 0.954)
	SMI	0.52 (0.308 - 0.732)
	L3-DRMI	0.756 (0.573 - 0.938)
Female	L3-SMHDR	0.975 (0.913 - 1)
	SMI	0.5 (0.194 - 0.806)
	L3-DRMI	0.938 (0.811 - 1)

SMI: skeletal muscle index, DRMI: density-related muscle index, SMHDR: skeletal muscle area adjusted to height and density ratio.

**Figure 2:**
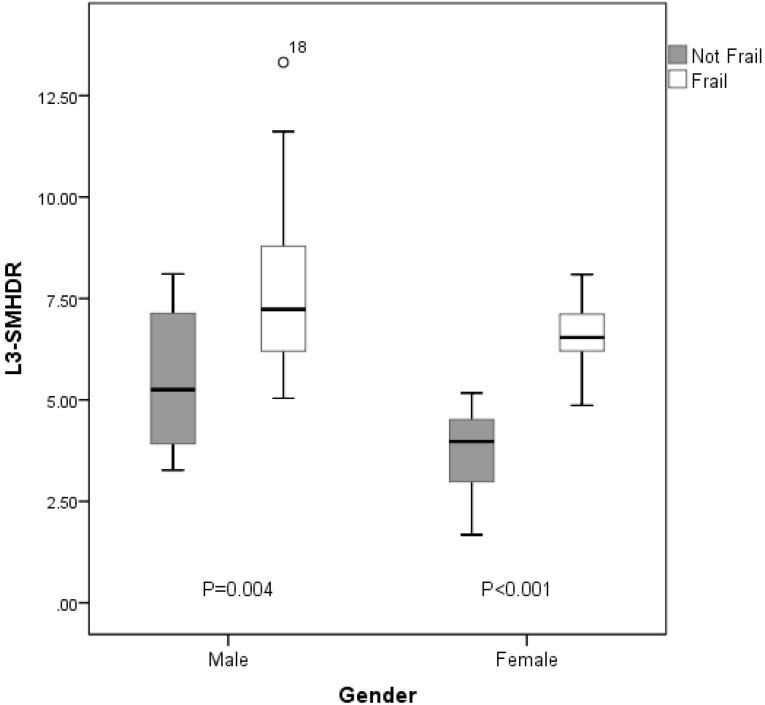
ROC curves for L3 SMI, L3-DRMI and L3-SMHDR depending on the sex

 The numeric parameter was then divided into two categories according to the respective sex-related cut-offs. Frailty was present in 79% of patients with higher than the cut-offs, compared with 21% with lower than the cut-offs (*P* < 0.001). This ratio with the divided categories has a sensitivity of 100%, a specificity of 76%, a positive predictive value of 79.3% and a negative predictive value of 100%. The L3-SMHDR was significantly correlated with frailty (r = 0.602, *P* < 0.001). The kappa score between frailty and L3-SMHDR was 0.752, with a percentage of agreement of 87.5%, showing a substantial agreement (Figure 2).

###  Prognostic Impact of L3-SMHDR

**Figure 3:**
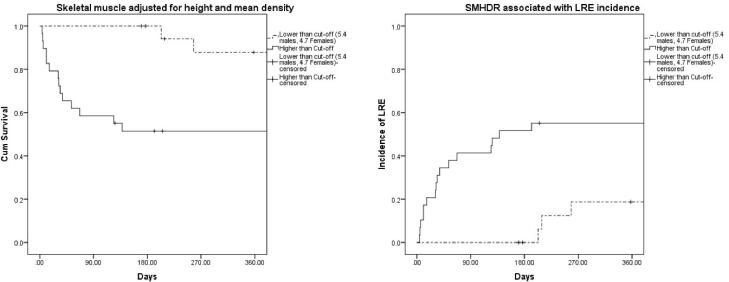
A- Survival time for patients with L3-SMHDR lower and higher than the cut-off (Log-rank Mantel-Cox *P* = 0.001) and B- Incidence of liver-related events with L3-SMHDR lower and higher than the cut-off (Log-rank Mantel-Cox *P* = 0.017)

 Survival analysis using L3-SMI with cut-offs of 50 for men and 39 for women did not show any statistically significant difference neither in terms of survival nor incidence of LRE (Log-rank Mantel-Cox P = 0.280 and 0.098, respectively). DRMI using cut-offs of 1.03 in men and 0.68 in women also showed a significant difference in terms of survival (Log-rank Mantel-Cox P = 0.04) but no significance for the incidence of LRE (Log-rank Mantel-Cox P = 0.068). The survival time was significantly lower in patients having L3-SMHDR higher than the cut-off, compared with patients having L3-SMHDR lower than the cut-off. The 90-day survival rate was 100% and 58.6% for, respectively for low L3-SMHDR and high L3-SMHDR (Log-rank Mantel-Cox P = 0.001). The one-year incidence of LRE was 18.7% and 55.2% for respectively low L3-SMHDR and high L3-SMHDR (Log-rank Mantel-Cox P = 0.017) (Figure 3).

## Discussion

 Diagnosis is essential because frailty is a dynamic and progressive diagnosis which can be prevented and may be reversible with an early diagnosis. The main treatment modalities that have been proven to improve frailty scores are physical exercise, nutrition and rehabilitation, with greater improvements after a combination of the three.^22^ Our study showed that cirrhotic frail patients had a lower total muscle density with higher intramuscular adipose tissue content. This is explained by the muscle breakdown triggered by the inflammatory cytokines, yielding amino acids that will be used for the production of energy.^23^ Thus, the increased demand for protein and increased inflammation will lead to disturbance of myocyte production and destruction. Our main findings were the L3-DRMI and L3-SMHDR and their association with frailty and prognosis in patients with cirrhosis. L3-DRMI and L3-SMHDR were both significantly associated with frailty in both male and female groups. L3-DRMI was shown to be associated with mortality but not the incidence of LRE.

 L3-SMHDR was significantly higher in child B/C patients with cirrhosiscompared with Child A patients. We set cut-offs for L3-SMHDR of 5.4 for men and 4.7 for women. Frailty was present in 79% of patients with higher than the cut-off and 21% with lower than the cut-offs. It is a score with a sensitivity and negative predictive value of 100%. This ratio is significantly correlated with frailty and has an impact on prognosis, including the survival time and incidence of LRE. We noted a significantly lower survival time in patients having L3-SMHDR higher than the cut-off.

 Several studies showed the association of CT parameters with the presence of sarcopenia and thus increased risk of frailty and mortality in patients with cirrhosis. In the US, we use the L3-SMI with a cut-off of less than 50 cm^2^/m^2^ in men and 39 cm^2^/m^2^ in women to diagnose sarcopenia in patients with cirrhosis and a correlation with waitlist mortality.^24^ Based on the Japanese guidelines, the L3-SMI cut-off values of less than 42 cm^2^/m^2^ for men and 38 cm^2^/m^2^ for women are used to diagnose sarcopenia.^25^ Another Japanese cohort showed an association between low SMI, high IMAC, high visceral to subcutaneous adipose tissue area ratio and increased mortality.^26^ The EASL suggests SMI cut-offs of 50 cm^2^/m^2^ for men and 39 cm^2^/m^2^ for women.^27^ Banjhi and others, using these cut-offs, identified the association with hepatic encephalopathy, regardless of the MELD score.^28^ Golse and colleagues used the psoas major area at the L3 or L4 level to predict the post-transplantation mortality after one year.^29^ Durand and co-workers showed that the transversal psoas muscle thickness normalized to height predicts mortality on the liver transplantation waiting list, independently of the MELD score.^30^

 The CT attenuation of the muscle reflects the quality of the skeletal muscle in patients with cirrhosis and, thus, the presence of myosteatosis. Frailty generally overlaps with sarcopenia and is associated with increased mortality.^31^ A study that included 106 compensated cirrhotic patients showed that the frailty diagnostic tests were correlated with CT-based muscle measures (muscle area and quality).^32^

 Our study identifies a new CT score directly related to the presence of frailty in patients with cirrhosis. The L3-SMHDR is a ratio with high sensitivity (100%), which means that all frail patients would be expected to have a L3-SMHDR higher than the cut-offs. Moreover, the high negative predictive value of 100% indicates that patients with a L3-SMHDR lower than the cut-offs are extremely unlikely to be frail. Moreover, frailty was inversely correlated with L3-SMHDR, and frailty and L3-SMHDR were correlated with LRE. This means that this score can be used to predict the occurrence of LRE in patients with cirrhosis. By using cut-offs for L3-SMHDR of 5.4 for men and 4.7 for women, this ratio could be used as a prognostic factor for frailty in cirrhosis because of its impact on quality of life and survival time.

 We note some limitations to our study. First, it is a single-center study with a limited number of patients; thus, the results may be affected by regional factors and may not be generalizable across all centers. Larger multicenter studies should be done to strengthen and validate the results. Second, the frailty scale risk assessment score relies on self-reported responses, which can lead to misinterpretation, overestimation or underestimation by patients.

 In conclusion, we identified predicting a CT score combining muscle mass and density for the prediction of frailty and identifying the prognostic implications in patients with cirrhosis, regardless of the disease etiology. Further large-scale studies are needed to confirm the reliability of this novel score for identifying frailty in cirrhosis and predicting short-term mortality and liver-related complications.
